# Urban carbon emissions: evolution, analysis, and application based on 53-year data

**DOI:** 10.3389/fpubh.2025.1713278

**Published:** 2026-01-13

**Authors:** Yang Ding, Kai-Jie Cao, Zhen-Zhen Guo, Jing-Liang Dong, Shuang-Xi Zhou, Zhi-Peng Lu

**Affiliations:** 1 Zhejiang Engineering Research Center of Intelligent Urban Infrastructure, Hangzhou City University, Hangzhou, China; 2 Department of Civil Engineering, Hangzhou City University, Hangzhou, China; 3 School of Civil Engineering and Architecture, East China Jiaotong University, Nanchang, China; 4 School of Civil and Engineering Management, Guangzhou Maritime University, Guangzhou, China; 5 School of Public Administration, Zhejiang Gongshang University, Hangzhou, China

**Keywords:** carbon emissions, urban sustainable development, data analysis, data prediction, policy analysis, time series analysis, urbanization

## Introduction

As one of the core drivers of global climate change, the accumulation and emission of carbon dioxide (CO_2_) in urban environments are profoundly affecting urban development from ecological, economic, and social dimensions, while also posing significant hazards (1–3). From a positive perspective, CO_2_ is a key raw material for plant photosynthesis. Rational green space planning in cities can utilize CO_2_ to promote vegetation growth, thereby enhancing urban ecological resilience and improving air quality (4). Meanwhile, with the development of low-carbon technologies, the resource-oriented utilization of CO_2_ has also provided a new direction for urban industrial upgrading, driving the rise of green industries such as new energy and environmental protection, and emerging as a potential growth point for urban economic transformation (5–7). However, its negative impacts and hazards are more prominent and far-reaching. Ecologically and environmentally, cities are high-density CO_2_ emission areas, and massive emissions have intensified the urban heat island effect—a phenomenon where urban center temperatures are significantly higher than those in surrounding suburbs. This not only increases energy consumption for cooling in summer but also may trigger extreme high-temperature weather, threatening residents' health (8, 9). Additionally, global climate anomalies caused by the greenhouse effect expose cities to higher risks of disasters such as frequent rainstorm-induced waterlogging and sea-level rise, undermining the safety of urban infrastructure (10). Economically, to address environmental issues caused by excessive CO_2_ emissions, cities need to invest huge funds in upgrading high-energy-consuming infrastructure, controlling pollution, and restoring ecosystems, which will increase fiscal pressure in the short term. Furthermore, the damage to urban lifeline systems caused by extreme climate disasters directly results in economic losses, affecting the normal operation of cities and their investment environment. Socially and in terms of public health, although high-concentration CO_2_ itself is non-toxic, it indirectly exacerbates urban air pollution (11–13). This may reduce air visibility and induce health problems such as respiratory diseases and cardiovascular diseases. Moreover, issues like agricultural output reduction and water scarcity caused by climate anomalies may indirectly affect the stability of urban food supply and intensify the pressure of social resource allocation (14–16). In summary, CO_2_ exerts a “dual-edged sword” effect on urban development. However, the hazards caused by current excessive emissions have far exceeded its limited positive role. Promoting urban low-carbon transformation and controlling CO_2_ emissions have thus become core tasks for ensuring the sustainable development of cities.

In the process of analyzing the impact of carbon emissions on urban development, the establishment of a carbon emission database holds irreplaceable significance. Firstly, by systematically collecting carbon emission data from various sectors within a city—including industry, construction, transportation, and residential life—the database can accurately identify high-emission areas and key sources of carbon emissions (17, 18). This provides a data foundation for targeted analysis of the specific harms caused by carbon dioxide emissions from different sectors to urban ecology, economy, and public health. Secondly, the functions of dynamic monitoring and trend analysis in a carbon emission database serve as core tools for assessing the evolutionary patterns of carbon emissions (19). Through long-term accumulation of carbon emission data, the database enables the construction of correlation models between carbon emissions and urban development (e.g., GDP), facilitating quantitative analysis of the corresponding relationship between changes in carbon emission intensity and urban development. It is evident that a carbon emission database is not only a measuring tool for quantifying carbon dioxide emissions but also a microscope for analyzing its urban impacts and a navigator for formulating governance plans (20). The level of its establishment and improvement directly determines the depth of understanding of urban development impacts and the effectiveness of response measures (21). It constitutes the core foundation for promoting the transformation of urban carbon governance from experience-based judgment to data-driven decision-making. Collectively, these studies establish the importance of emission driver analysis but suffer from two gaps: (1) reliance on fragmented or short-term data, and (2) insufficient attention to intra-urban regional differences.

To mitigate the hazards of excessive carbon dioxide (CO_2_) emissions on urban development, China has implemented a series of targeted technological measures and built a multi-dimensional carbon reduction technology system, focusing on key areas such as energy structure optimization, industrial upgrading, and infrastructure renovation. For instance, on the supply side, China has vigorously promoted the application of renewable energy substitution technologies. Within urban areas, clean energy power generation systems—including photovoltaic (PV), wind, and biomass energy—have been widely deployed (22–24). Meanwhile, ultra-high voltage (UHV) power transmission technology is used to deliver cross-regional clean energy to cities, gradually reducing urban reliance on thermal power and cutting CO_2_ emissions related to energy consumption at the source. In the construction sector, green building technologies have been fully popularized (25). Through new thermal insulation materials, passive building designs, and renewable energy-based heating and cooling technologies, energy consumption throughout the entire life cycle of buildings has been reduced. At the same time, energy-saving retrofits of existing buildings have been advanced to improve the energy efficiency of established structures. These technological measures work in synergy to drive urban carbon reduction across the entire chain of source control, emission reduction, recycling, and sequestration (26, 27). They provide core technological support for alleviating the hazards of CO_2_ on urban development and realizing low-carbon, sustainable urban development.

Meanwhile, China put forward the Dual Carbon goals in 2020, namely achieving carbon peaking by 2030 and carbon neutrality by 2060 (28). To this end, a series of policies have been formulated. For example, the 2024–2025 Action Plan for Energy Conservation and Carbon Reduction issued by the State Council emphasizes the following key measures: Strictly controlling coal consumption, advancing the low-carbon transformation of coal-fired power and the triple renovation coordination, and cutting non-electric coal use (29). In key regions for air pollution prevention and control, newly built, renovated, and expanded coal-consuming projects shall implement coal substitution with equivalent or reduced quantities. Vigorously developing large-scale wind and photovoltaic power bases, with a focus on deserts, Gobi, and arid areas; developing offshore wind power in a rational and orderly manner; and promoting the utilization of distributed new energy (30, 31). Advancing the low-carbon transformation of transportation infrastructure and encouraging the construction of green highways and railways. Promoting the shift of transportation equipment to new energy, advancing the electrification of vehicles in the public sector, and developing zero-emission freight fleets (32, 33). For newly built buildings, requiring that by the end of 2025, all newly built urban buildings fully comply with green building standards; increasing the coverage rate of photovoltaic systems on the rooftops of newly built public institution buildings and factory buildings; raising the renewable energy substitution rate for urban buildings; and expanding the floor area of newly built ultra-low energy consumption and near-zero energy consumption buildings (34, 35). Specially, Hangzhou epitomizes the evolutionary trajectory of China's large and medium-sized cities, particularly in balancing rapid urbanization, economic growth, and carbon emission control, a core challenge faced by global emerging economies in the context of climate change. And Hangzhou's industrial composition uniquely encapsulates the dual characteristics of traditional high-emission sectors and emerging low-carbon industries, a key tension in global urban low-carbon transformation.

Based on the above, this study mainly takes the nearly 50-year carbon emission data of Hangzhou, Zhejiang Province—specifically the data from 1970 to 2023—as the foundation. It analyzes the correlation between urban development (including urban population and urban GDP) and carbon emissions, and studies the impact of carbon reduction policies on carbon emission levels. Secondly, the study constructs a carbon emission prediction model based on artificial intelligence technology. This model not only enables carbon emission prediction across various districts and counties but also further establishes a predictive model between the carbon emissions of individual districts/counties and the total urban carbon emissions. Finally, integrating existing carbon reduction policies, the study proposes new carbon reduction strategies, such as the establishment of carbon emission rights and their corresponding markets, and the formulation of gradient carbon pricing mechanisms.

## Analysis of carbon emission data in Hangzhou

**Figure 1:**
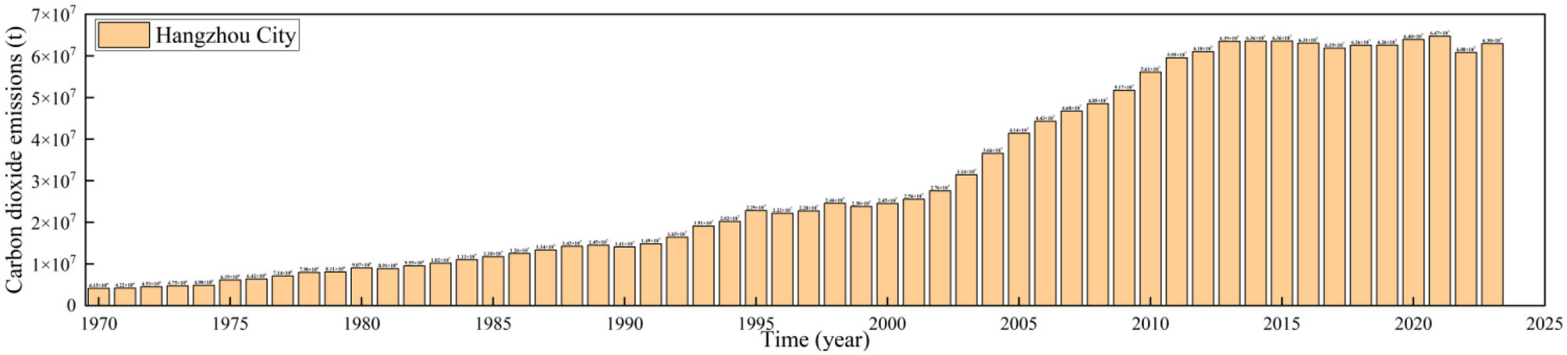
Carbon emission data of Hangzhou City.

The total carbon emissions of Hangzhou from 1970 to 2023 are shown in Figure 1. As can be seen from the figure, the total carbon emissions of Hangzhou exhibited a rapid growth trend. Specifically, the total carbon emissions of Hangzhou stood at 4,152.27 × 103 tons in 1970, while by 2023, this figure had reached 62,967.01 × 103 tons, representing a 15.16-fold increase. Meanwhile, according to reports from the National Bureau of Statistics, Hangzhou's GDP was approximately 2 billion RMB (0.002 trillion RMB) in 1970, with a total registered population of 4.6 million and a total urban population of 1.0194 million. By 2023, Hangzhou's GDP had grown to around 2.01 trillion RMB, and its permanent resident population had reached 12.522 million. It is evident that with the rapid advancement of urbanization, Hangzhou's carbon emissions have increased sharply. The main reasons for this are as follows: (1) During the urbanization process, the development of industry and the expansion of industrial land have led to a significant rise in carbon emissions; (2) As urbanization progresses, the growth of the urban population has increased demand for transportation, and the continuous expansion of motor vehicle ownership has caused a rapid surge in carbon emissions from the transportation sector; (3) In the course of urbanization, a large number of buildings have been constructed and put into use. Energy consumption during building construction, as well as demands for heating, cooling, and lighting during building operation, have all contributed to increased carbon emissions; and (4) The growth of population and the rise in urbanization rate have brought about rigid growth in energy consumption and carbon emissions. At the same time, improvements in residents' living standards and the upgrading of consumption have increased household energy consumption, further driving up carbon emissions. While 1970 population data are based on registered residents and 2023 data on permanent residents, the National Bureau of Statistics estimates that Hangzhou's 1970 permanent resident population was approximately 4.6 million, minimizing bias in long-term growth analysis

In addition, in September 2020, China explicitly put forward the goals of achieving carbon peaking by 2030 and carbon neutrality by 2060. The introduction of this policy has also significantly slowed the growth of Hangzhou's total carbon emissions. Specifically, Hangzhou's carbon emissions were 63,960.34 × 103 tons in 2020, 64,737.12 × 103 tons in 2021, 60,843.66 × 103 tons in 2022, and 62,967.01 × 103 tons in 2023, as shown in Figure 1. Specifically, the raw carbon emission data is attached in the Appendix of this article. It is evident that after responding to the national policy, Hangzhou's carbon emissions have tended to stabilize. The measures adopted are as follows: (1) Relying on the construction of its City Brain, Hangzhou has built a four horizontal and three vertical intelligent governance framework for the Dual Carbon goals, establishing a full-chain governance system covering the government, enterprises, and individuals; (2) Implementing infrastructure projects such as distributed photovoltaic power generation and centralized heating in industrial parks to achieve cascaded energy utilization; and (3) Integrating high-frequency monitoring data from sectors including electricity and industry through the energy Dual Carbon digital and intelligent platform, establishing carbon emission accounting models and corporate carbon accounts, and realizing hierarchical management of energy budgets and carbon budgets.

**Figure 2:**
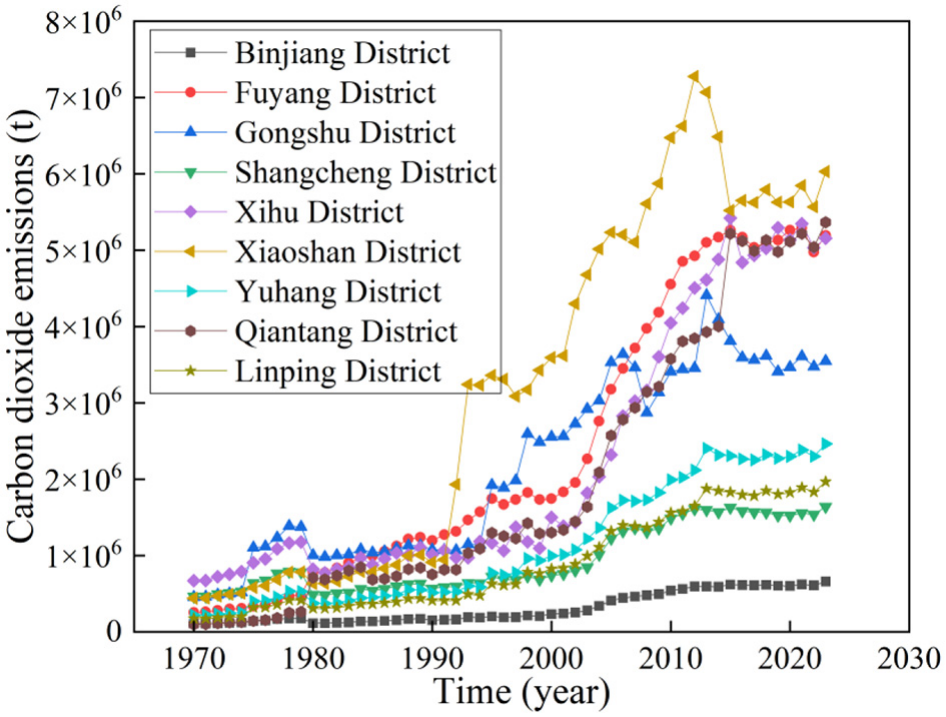
Carbon emission data of various districts in Hangzhou.

Further, carbon emission data from several districts under Hangzhou—including Binjiang District, Fuyang District, Gongshu District, Shangcheng District, Xihu District, Xiaoshan District, Yuhang District, Qiantang District, and Linping District—from 1970 to 2023 are presented in Figure 2. As shown in Figure 2, the growth trend of carbon emissions in each district is similar to that of Hangzhou's total carbon emissions: in the early stage, with the acceleration of urbanization, carbon emissions in each district increased rapidly; later, following the issuance of the Dual Carbon policies, carbon emissions in each district stabilized or even showed a downward trend. Specifically, in 2023, Xiaoshan District had the highest carbon emissions, reaching 6,029.51 × 103 tons, while Binjiang District had the lowest, at 658.42 × 103 tons. This is mainly due to the different functional orientations of the two districts. For example, Xiaoshan District has the strongest industrial foundation in Hangzhou, hosting national-level industrial platforms such as the Xiaoshan Economic and Technological Development Zone. It focuses on developing high-energy-consuming industries including automobile manufacturing, mechanical equipment, textile printing and dyeing, and chemical engineering. Additionally, Xiaoshan International Airport is a key aviation hub in East China: in 2023, its passenger throughput exceeded 40 million, and cargo throughput reached 8,00,000 tons, with significant carbon emissions from aviation fuel consumption and logistics transportation. Meanwhile, the district has dense logistics parks and a high proportion of road freight, which further drives up emissions in the transportation sector. In contrast, Binjiang District centers on the digital economy. In 2023, the added value of its core digital economy industries accounted for 75.6% of its GDP. It has formed emerging industrial clusters in the Internet of Things (IoT), artificial intelligence (AI), and biomedicine, led by Internet giants such as Alibaba and NetEase. The energy consumption intensity of these industries is far lower than that of traditional industries. Additionally, in 2021, Hangzhou adjusted the administrative divisions of some districts: the new Shangcheng District covers the original administrative areas of the former Shangcheng District and Jianggan District; the new Gongshu District covers the original administrative areas of the former Xiacheng District and Gongshu District.

## Carbon emission prediction

As a core district of Hangzhou, Xiaoshan maintains intensive economic and industrial linkages with neighboring districts—for instance, the spillover of manufacturing activities and energy consumption between Xiaoshan and Binjiang, Gongshu, and Yuhang districts directly affects their mutual carbon emission dynamics. Furthermore, this article uses the carbon emissions of eight adjacent regions to predict the emissions of Xiaoshan, with the goal of capturing the spatial spillover effects of interrelated regional development patterns and regional level carbon emissions.

Combined with the artificial neural network (ANN) method, the carbon emission data of Binjiang District, Fuyang District, Gongshu District, Shangcheng District, Xihu District, Yuhang District, Qiantang District, and Linping District were used as input, while the carbon emission data of Xiaoshan District were used as output to analyze the impact of the prediction horizon on the model's prediction performance. Specially, MLP was selected because it effectively models the nonlinear relationships between multi-source regional emission data without imposing strict assumptions on data distribution, which is suitable for our cross-district correlation analysis. The hidden layers use the Rectified Linear Unit (ReLU) function, which addresses the vanishing gradient problem during training and accelerates convergence. The output layer adopts a linear activation function to accommodate the continuous nature of carbon emission values. The final model analyzed the impact of hidden layers. Simultaneously, training set is 90% of total data and Test set is 10% of total data and time-Series 5-Fold Cross-Validation to assess model stability. Due to the time-varying nature of the data, this paper did not consider the validation set.

**Figure 3:**
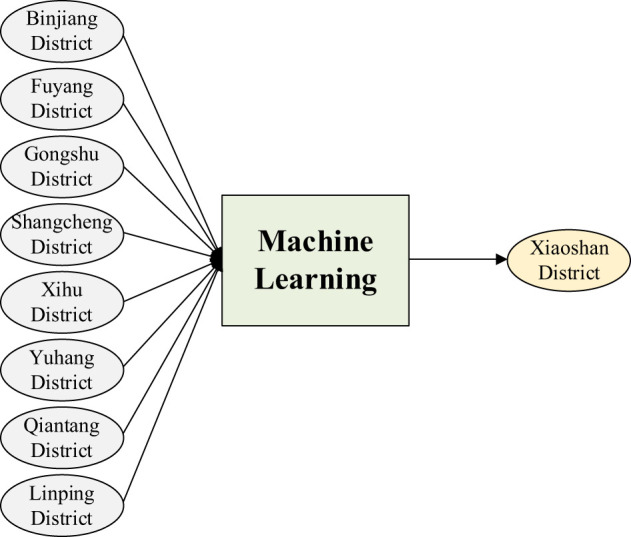
Prediction model structure.

**Figure 4:**
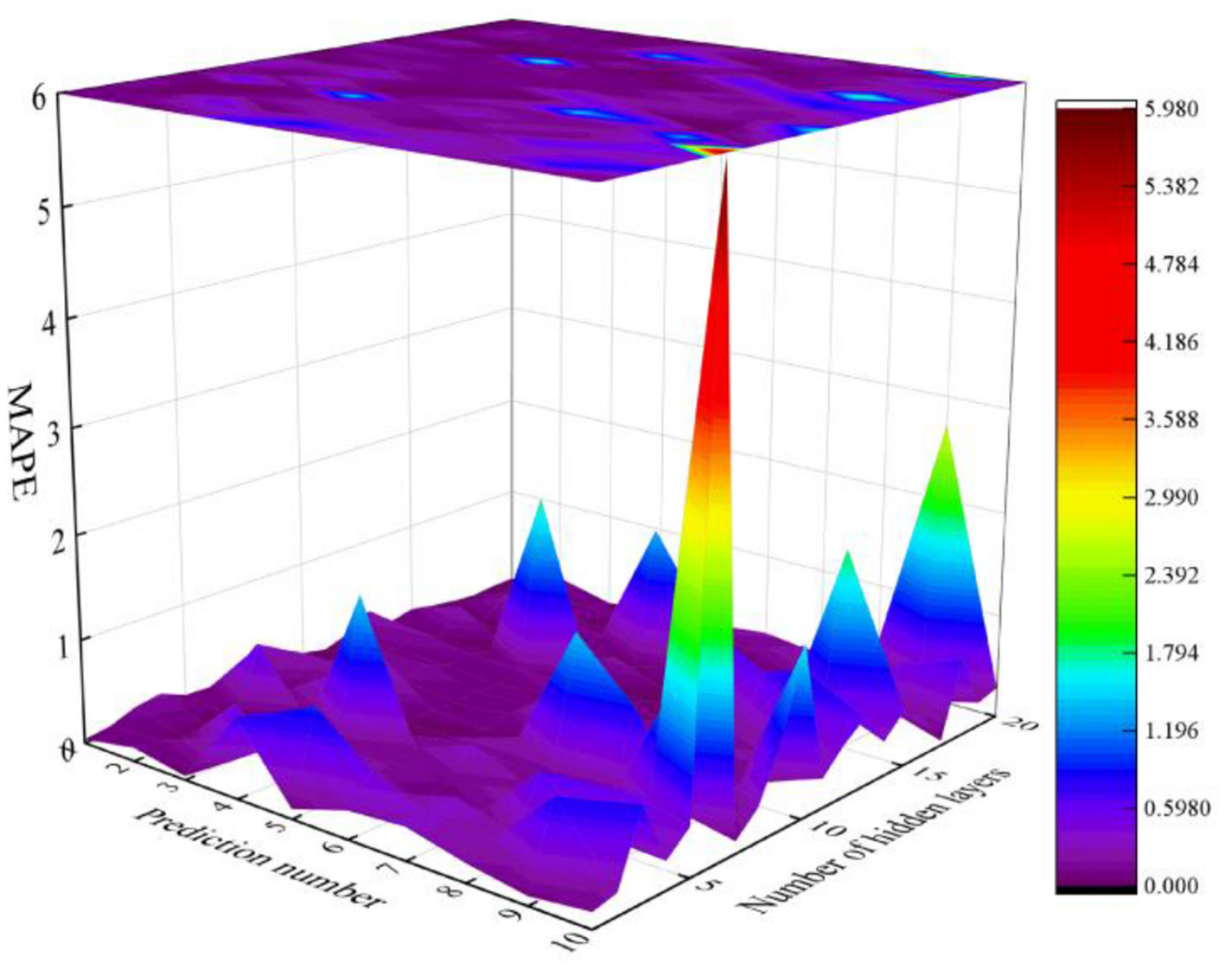
The relationship between predictive performance, prediction quantity, and model structure.

The prediction model is shown in Figure 3. Among them, the Mean Absolute Percentage Error (MAPE) is used as a performance evaluation metric. As the number of predicted years increases, the overall predictive performance of the model shows a downward trend. For example, when the number of hidden layers is 20, the MAPE value predicted for 1 year is 0.03163; The MAPE value predicted for 2 years is 0.18401; The MAPE value predicted for 3 years is 0.05943; The MAPE value predicted for 4 years is 0.13721; The MAPE value predicted for 5 years is 0.05372; The MAPE value predicted for 6 years is 0.20275; The MAPE value predicted for 7 years is 0.13409; The MAPE value predicted for 8 years is 0.44268; The MAPE value predicted for 9 years is 2.72291; The MAPE value predicted for 10 years is 0.28661, as shown in Figure 4.

Meanwhile, when the number of predictions is the same, the prediction performance fluctuates with the increase of the number of hidden layers. For example, when the predicted number is 1 year and the number of hidden layers is 1, the MAPE is 0.03548; When the number of hidden layers is 2, the MAPE is 0.07236; When the number of hidden layers is 3, the MAPE is 0.16655; When the number of hidden layers is 4, the MAPE is 0.20551; When the number of hidden layers is 5, the MAPE is 0.10015; When the number of hidden layers is 6, the MAPE is 0.08698; When the number of hidden layers is 7, the MAPE is 0.14562; When the number of hidden layers is 8, the MAPE is 0.3485; When the number of hidden layers is 9, the MAPE is 0.20822; When the number of hidden layers is 10, the MAPE is 0.07561; When the number of hidden layers is 11, the MAPE is 0.02716; When the number of hidden layers is 12, the MAPE is 0.10328; When the number of hidden layers is 13, the MAPE is 0.26043; When the number of hidden layers is 14, the MAPE is 0.05878; When the number of hidden layers is 15, the MAPE is 0.10927; When the number of hidden layers is 16, the MAPE is 0.06443; When the number of hidden layers is 17, the MAPE is 0.02863; When the number of hidden layers is 18, the MAPE is 0.00364; When the number of hidden layers is 19, the MAPE is 0.02547; When the number of hidden layers is 20, the MAPE is 0.03163. It can be seen that the predictive performance of BP neural network will fluctuate to some extent under different hidden layers and different prediction quantities, indicating poor robustness. However, for the prediction of carbon emissions in Hangzhou, multiple predictions can be averaged to reduce prediction errors; At the same time, errors can be reduced by debugging the number and layers of hidden layers.

Clearly, the MAPE values in Figure 4 fluctuate greatly, the reasons can be explained as: First, dynamic changes in spatial economic-industrial linkages between the 8 input districts and Xiaoshan, for instance, strengthened spillover effects from Binjiang's digital economy expansion (2018–2020) reduced MAPE, while Fuyang's 2021 textile policy adjustments weakened cross-district correlations and increased MAPE. Second, the sensitivity of the MLP model to hidden layer configuration: too few layers led to underfitting, while excessive layers caused overfitting to data noise. Third, time-scale-dependent uncertainty: short-term predictions (1–3 years) benefited from stable industrial/policy conditions, while medium-to-long-term forecasts (4–10 years) were disrupted by unforeseeable factors, leading to spikes like the 9-year MAPE of 2.72291. Fourth, structural breakpoints in historical data−1990's shift from self-calculated to official data and 2020's Dual Carbon Goals-induced emission trend change—introduced subtle data heterogeneity that propagated to predictions.

## Research on carbon emission policies

As an emerging environmental right, carbon emission rights serve as the cornerstone for building a carbon emission market (36). Amid the global effort to address climate change, China has actively promoted the development of a carbon emission rights trading system. By setting scientific and reasonable total carbon emission targets, emission rights are allocated to key emitting enterprises in the form of quotas. These enterprises can trade their quotas in the market based on their own emission reduction performance. This market-oriented mechanism incentivizes enterprises with low emission reduction costs to exceed their reduction targets and sell surplus quotas for profit. Conversely, enterprises facing high difficulties in emission reduction can purchase quotas to meet compliance requirements. In this way, the mechanism not only stimulates enterprises' initiative to reduce emissions independently but also achieves the overall emission reduction target at a lower social cost. Specifically, the annual total carbon emission quotas are formulated in a scientific and reasonable manner, taking into account factors such as the industrial development stage, historical emission data, future emission reduction potential, and the needs of economic and social development. In the initial stage, quota allocation is dominated by free distribution, with appropriate inclination toward high-energy-consuming, high-emission industries that face significant challenges in emission reduction—this ensures a stable industrial transition. As the market matures, the proportion of paid allocation will be gradually increased to enhance the efficiency and fairness of quota allocation. Meanwhile, a quota reserve mechanism will be established to respond to sudden market fluctuations and stabilize market expectations.

Gradient carbon pricing is an innovative price regulation tool that plays a key role in optimizing the allocation of carbon emission resources. The traditional single carbon price struggles to fully reflect differences in the marginal abatement costs and emission reduction potential among different enterprises. In contrast, gradient carbon pricing sets different price ranges based on enterprises' carbon emission levels. When an enterprise's carbon emissions are at a low level, a lower carbon price applies—this encourages the enterprise to maintain and further reduce its emissions. For high-emission enterprises, however, as their emissions exceed the specified threshold, the carbon price increases significantly and progressively. This raises their emission costs, creating a strong constraint. This mechanism compels enterprises to fully consider carbon emission factors when formulating production decisions and technological innovation strategies, guiding resources to flow toward low-carbon and green sectors. For example, in some pilot regions, after the implementation of gradient carbon pricing, high-energy-consuming enterprises have accelerated the technological transformation for energy conservation and emission reduction; some enterprises have even proactively adjusted their industrial structure to transition to low-carbon industries, effectively driving the green development of the regional economy.

Overall, Xiaoshan's emissions are dominated by energy-intensive industries, that is, automobile manufacturing, chemical engineering, textile printing and dyeing, and air logistics, that is, Xiaoshan International Airport's passengers over 40 million and 8,00,000 tons of cargo in 2023. Enterprises exceeding this threshold would face a progressive carbon price increase, e.g., 10% premium for 10–20% overshoot, 30% premium for over 20% overshoot. This directly aligns with Hangzhou's post-2020 emission stabilization by incentivizing Xiaoshan's high-emission sectors to reduce emissions without disrupting industrial transition. For carbon emission rights trading, it suggest that connect the free initial allocation + gradual paid allocation mechanism to Xiaoshan's industrial structure. Initially, 80% of quotas are freely allocated to Xiaoshan's key high-emission enterprises to ensure stable operation during low-carbon transformation. As the market matures, the paid allocation ratio will increase to 30% by 2030, encouraging enterprises to optimize energy efficiency. For Binjiang District, surplus quotas from low-emission IoT/AI enterprises can be traded to Xiaoshan's enterprises, creating a district-level carbon market that supports Hangzhou's stabilization goals.

## Conclusions

The paper's core objective is decomposed into three specific, measurable sub-objectives: Quantify the 1970–2023 evolutionary trends of Hangzhou's carbon emissions and identify key drivers by linking emissions to urban development indicators and comparing regional disparities; Develop an Artificial Neural Network (ANN) model that captures cross-district spatial spillover effects, using emissions from 8 districts as input and Xiaoshan's as output to support district/city-level prediction, with optimization; Propose regionally differentiated policies, including a free initial allocation + gradual paid allocation carbon quota mechanism for Xiaoshan's high-emission sectors and gradient pricing to incentivize reduction, while enabling Binjiang's surplus quotas to be traded to Xiaoshan

And the paper's innovations lie in: Leveraging 53 years of continuous monitoring data to analyze long-term emission-driver relationships across urbanization stages; Designing a cross-district ANN model to overcome the limitation of single-region prediction and reflect inter-district economic/industrial linkages; Developing targeted policies that avoid one-size-fits-all frameworks by aligning with district-specific industrial characteristics, bridging the gap between macro goals and micro implementation.

## Data Availability

The original contributions presented in the study are included in the article/Supplementary material, further inquiries can be directed to the corresponding author.
